# Prevalence of HPV Positivity and the Correlation Between P16INK4A Expression and HPV DNA Positivity in Carcinoma Oropharynx and Their Correlation With Survival Outcomes: A Retrospective Study From a Tertiary Cancer Centre in South India

**DOI:** 10.7759/cureus.77162

**Published:** 2025-01-08

**Authors:** Amitha Sherief P, Lekha Madhavan Nair, Rejnish Ravikumar, Preethi Sara George, Kainickal Cessal Thommachan, Malu Rafi, Lakshmi S, Devasena Anantharaman, Radhakrishna Pillai M, Kunnambath Ramadas

**Affiliations:** 1 Medical Oncology, Regional Cancer Centre, Thiruvananthapuram, IND; 2 Radiation Oncology, Regional Cancer Centre, Thiruvananthapuram, IND; 3 Epidemiology and Biostatistics, Regional Cancer Centre, Thiruvananthapuram, IND; 4 Cancer Research, Regional Cancer Centre, Thiruvananthapuram, IND; 5 Biotechnology, Rajiv Gandhi Centre for Biotechnology, Thiruvananthapuram, IND; 6 Radiation Oncology, Clinical Operations and Allied Services, Karkinos Healthcare, Thiruvananthapuram, IND

**Keywords:** carcinoma oropharynx, hpv dna, human papillomavirus, p16 ihc, survival

## Abstract

Introduction

The incidence of oropharyngeal squamous cell carcinoma (OPSCC) has been increasing worldwide. High-risk human papillomavirus (HPV) infection is now a well-recognised risk factor for oropharyngeal cancers. However, the information regarding the prevalence and outcome of HPV-related OPSCC is sparse in India. The study was conducted to identify the frequency of HPV infection in oropharyngeal cancer and also to study the treatment response and survival according to HPV positivity and p16^INK4A^ expression.

Materials and methods

The study sample consists of 100 paraffin-embedded tissue blocks of histologically proven OPSCC patients who had undergone treatment at a tertiary cancer centre in Kerala, India, from January 2010 to December 2012. The patients' medical records were examined to obtain demographic data, information on habits, and clinical, histopathological, and treatment information. Follow-up information on disease status and vital status was collected until May 2023. Paraffin-embedded tissue blocks of these patients were collected from the archives of the Division of Pathology. Immunohistochemistry (IHC) was used to identify p16 expression. HPV DNA was isolated from the paraffin-embedded tissue blocks by polymerase chain reaction.

Statistical analysis and results

Survival curves were obtained using the Kaplan-Meier method and compared with the log-rank test. The influence of p16 status and HPV DNA positivity on survival and recurrence was assessed using Cox regression. A total of 100 patients diagnosed with oropharyngeal malignancy and their paraffin-embedded blocks were used for the present study. p16 IHC was invalid for three patients, and 16 patients had invalid HPV DNA. Two patients were excluded from survival analysis because they had both invalid HPV DNA and p16 expression. A total of 98 patients were included in the analysis. Out of 98 samples assessed, 47 tested positive for p16 expression, 48 were negative, and three showed invalid results. Among the 98 patients, HPV DNA results were available for 82 patients. HPV DNA positivity was reported in 25 patients, and 57 samples were HPV negative. There was no significant correlation between p16 expression and HPV status. The median follow-up was 134 months (1-160 months). The five-year overall survival (OS) probability was 42.6% (95% confidence interval (CI) 28.49-56.71) and 51.2% (95% CI 35.92-66.48), respectively, for p16-negative and p16-positive tumours (p=0.689). The corresponding figures for five-year disease-free survival (DFS) were 49.0% (95% CI 34.7-63.3) and 51.9% (95% CI 36.62-67.18), p=0.959. The five-year OS for HPV DNA-negative tumours was 45.5% (95% CI 32-59.02) compared to 49.1% (95% CI 28.72-69.48) in HPV DNA-positive tumours. There was an absolute difference of 20% in five-year OS between double-positive and double-negative tumours.

Conclusion

This study demonstrated a p16 positivity rate of 49.47% and an HPV DNA positivity rate of 30.37%. However, only 15.18% of cases showed double positivity. No significant correlation was observed between p16 expression and HPV status. Double positivity (p16 and HPV positive) was associated with better OS and DFS compared to double-negative (p16 and HPV negative) and single-positive (either p16 positive or HPV positive) cases. This subgroup of patients might benefit from potential de-escalation strategies and should be the target population for future studies.

## Introduction

Head and neck cancer is the seventh most common cancer worldwide, with an estimated annual incidence of 888,000 cases [1]. The overall incidence of head and neck squamous cell carcinoma (HNSCC) has decreased in the last three decades; however, the incidence of oropharyngeal squamous cell carcinoma (OPSCC), mainly in the tonsil and base of the tongue, has been increasing across the globe [2]. High-risk human papillomavirus (HPV) infection is now a well-recognised risk factor for HNSCC, especially oropharyngeal cancers [3]. The increased incidence of HPV-related HNSCC mostly occurs in individuals aged between 40 and 55 years without a history of tobacco and alcohol consumption [4]. Several studies have demonstrated a change in sexual behavioural patterns, which has contributed to the rise in HPV-related oropharyngeal cancer in the younger population [5,6].

Several studies have shown better disease-free survival (DFS) and overall survival (OS) in HPV-associated OPSCC [7,8]. There is a separate AJCC (American Joint Committee on Cancer) staging system for HPV-positive OPSCC, and there is growing support for the view that treatment protocol for oropharyngeal cancer should be modified according to HPV status. Several trials on the de-escalation of treatment in HPV-positive OPSCC have been published, and many are underway.

Most of the published literature on the role of HPV in OPSCC is from the Western world. The majority of the head and neck cancers in India are tobacco-related. Moreover, the genetic makeup, cultural practices, and behavioural patterns are different compared to the Western population. The information regarding the association of HPV and OPSCC is sparse in India. The objectives of this study are (1) to identify the frequency of HPV infection in oropharyngeal cancer patients reported to the tertiary cancer centre in South India, (2) to study the correlation between HPV positivity and p16^INK4A^ expression, and (3) to study the treatment response and survival of HPV-related OPSCC. This article was previously presented as a scientific poster at the ESMO Asia Congress on 7th December 2024.

## Materials and methods

The study sample consists of 100 paraffin-embedded tissue blocks of histologically proven OPSCC patients who had undergone treatment at a tertiary cancer centre in Kerala, India, from January 2010 to December 2012. The study was conducted after obtaining approval from the Institutional Review Board (IRB/11-2012) and Human Ethics Committee (HEC No: 36/2011). Patients with histologically proven SCC of the oropharynx who underwent treatment at the centre during the study period were included in the study. Patients whose paraffin blocks were unavailable and those with no tumour tissue in the paraffin blocks were excluded from the study. Medical records of the patients were examined to obtain demographic data, information on habits, and clinical, histopathological, and treatment information. The staging was done according to the AJCC 7th edition TNM staging. Follow-up information on disease status and vital status was collected until May 2023 using data from medical records and through active telephonic contact. Paraffin-embedded tissue blocks of these patients were collected from the archives of the Division of Pathology.

HPV DNA was isolated from the paraffin-embedded tissue blocks by polymerase chain reaction (PCR). The multiplex PCR was used to amplify the targeted sequences using HPV type-specific primers targeting the E7 region for the detection of 19 high-risk (HR)/probable HR-HPV types (16, 18, 26, 31, 33, 35, 39, 45, 51, 52, 53, 56, 58, 59, 66, 68a, 68b, 70, 73, 82) and two low-risk (LR)-HPV types (HPV 6 and 11), with detection limits ranging from 10 to 1000 copies of the viral genome. Primers specific for the amplification of the housekeeping gene beta globin were included in the primer mix to provide a positive control for the quality of the template DNA. The amplified products were stored at -20°C until further processing. The presence of HPV-16 DNA or HPV-18 DNA or both was taken as HPV positive.

To identify p16 expression by IHC, 4 μm thick sections of the paraffin-embedded tissue samples were taken on poly L-lysine-coated slides. The primary and secondary antibodies used were *CDKN2A*/p16^INK4a^ antibody [EPR1473] from Abcam and super sensitive PolyExcel Poly HRP (PolyExcel Detection System, PathnSitu, Pleasanton, USA), respectively. The antibody staining was visualised using an Axioscope 2 Plus (Leica, Wetzlar, Germany) microscope, and images were captured using Canon Zoom Browser EX, Melville, USA. A known cervical sample positive for p16^INK4A^ expression was taken as a positive control. Four randomly selected areas of the stained sections were analysed for evaluating staining at 40× magnification. The p16^INK4A^ immunostaining was considered positive when either the nucleus or cytoplasm or both showed positive staining. The staining was scored as follows: negative (<10% of cells positive), mild (<20% of cells positive), moderate (20-50% of cells positive), and intense (>50% of cells positive). For statistical analysis, mild staining is grouped as a negative expression, and moderate and intense staining is grouped as a positive expression.

Statistical analysis

Continuous variables were described using descriptive statistics (mean, median, standard deviation, minimum, and maximum). The differences in categorical variables between groups were evaluated using the chi‑square test and the difference in continuous variables using the two‑sample t‑test. OS was calculated from the date of diagnosis to the date of either death or the last follow-up. Disease-free survival was calculated from the date of diagnosis to the date of recurrent disease, death, or date of last follow-up. Survival curves were obtained using the Kaplan‑Meier method and compared with the log‑rank test. Kaplan-Meier curves are used routinely to estimate and visualise the survival probabilities over time for different groups within a population. The survival curves help compare survival outcomes between patients with varying expressions of p16 or HPV DNA status in this study.

The method accounts for right-censored data (patients lost to follow-up or still alive at the end of a study), ensuring that the analysis is unbiased. The influence of p16 status and HPV DNA positivity on survival and recurrence was assessed by Cox regression. Cox regression is a statistical technique used to identify the relationship between the survival time of the patients and one or more predictor variables, such as HPV status, p16 expression, age, gender, and other clinical factors. It estimates the hazard ratio, which indicates the risk of an event (e.g., death, recurrence) for a particular variable compared to a baseline group. Statistical analysis was done using IBM SPSS Statistics for Windows, Version 28 (Released 2021; IBM Corp., Armonk, New York). Any p-value less than 0.05 was considered statistically significant.

A total of 100 patients diagnosed with oropharyngeal malignancy and their paraffin-embedded blocks were used for the present study. Three patients exhibited invalid p16 IHC, and 16 patients had invalid HPV DNA. They were not considered for correlation analysis but were included for survival analysis. Two patients were also excluded from survival analysis because they had both invalid HPV DNA and p16 expression.

## Results

A total of 98 patients were included in the analysis, with 90 males and eight females. The median age was 58 years. The baseline patient characteristics are shown in Table 1.

**Table 1:** Baseline patient characteristics BOT: base of the tongue; WD: well-differentiated; MD: moderately differentiated; PD: poorly differentiated; SCC: squamous cell carcinoma; NOS: not otherwise specified

Variable			Frequency (%) N=98
Gender	Male		90 (91.8)
	Female		8 (8.2)
Smoking	Yes		79 (80.6)
	No		19 (19.4)
Chewing	Yes		18 (18.4)
	No		80 (81.6)
Alcoholism	Yes		68 (69.4)
	No		30 (30.6)
Subsite		Tonsil	49 (50)
		BOT/valleculae	32 (32.6)
		Soft palate	11 (11.2)
		Pharyngeal wall	6 (6.1)
Histology	WD SCC		1 (1.02)
	MD SCC		43 (43.87)
	PD SCC		24 (24.5)
	SCC NOS		30 (30.6)
T stage	T1		10 (10.2)
	T2		25 (25.5)
	T3		42 (42.9)
	T4		21 (21.4)
N stage	N0		23 (23.5)
	N1		30 (30.6)
	N2		37 (37.8)
	N3		8 (8.2)
M stage	M0		97 (99)
	M1		1 (1)
Composite stage	I		4 (4.1)
	II		2 (2.0)
	III		39 (39.8)
	IV		53 (54.1)

Out of 98 samples assessed, 47 tested positive for p16 expression, 48 were negative, and three showed invalid results. Among the 98 patients, HPV DNA results were available for 82 patients. HPV DNA positivity was reported in 25 patients, and 57 were HPV negative. Among the 25 HPV-positive patients, 16 were HPV-16 positive, 14 patients tested positive for HPV-18, and five of them were both positive. The correlation of p16 status and HPV status with gender, habits, histological subtypes, stage, and subsite was performed using the chi‑square test (Table 2).

**Table 2:** Correlation of p16 and HPV status with patient and tumour characteristics SD: standard deviation; BOT: base of the tongue; IHC: immunohistochemistry; HPV: human papillomavirus; DNA: deoxyribonucleic acid; WD: well-differentiated; MD: moderately differentiated; PD: poorly differentiated; SCC: squamous cell carcinoma; NOS: not otherwise specified

Variables		p16 IHC Tested			HPV DNA Tested		
		Overall sample n=95	p16 positive n=47	p-value	Overall sample n=82	HPV positive n=25	p-value
Age, years median (SD)		58.0 (10.2)	58.0 (10.8)	0.991	57 (10.6)	55.0 (10.4)	0.994
Age (years)	≤50	20	10 (50)	0.960	57	7 (28.0)	0.246
	>50	75	37 (49.33)		25	18 (72.0)	
Gender	Female	8	3 (37.5)	0.471	5	2 (40.0)	0.631
	Male	87	44 (50.6)		77	23 (23.9)	
Tobacco smoking	No	19	8 (42.1)	0.472	15	5 (33.3)	0.795
	Yes	76	39 (51.3)		67	20 (29.9)	
Chewing	No	77	41 (53.2)	0.128	67	22 (32.8)	0.327
	Yes	18	6 (33.3)		15	3 (20.0)	
Alcohol use	No	30	16 (53.3)	0.610	25	6 (24.0)	0.395
	Yes	65	31 (47.7)		57	19 (33.3)	
Subsite	Tonsil	47	26 (55.3)	0.0001	42	17 (40.5)	0.001
	BOT/vallecula	32	16 (50.0)		27	5 (18.5)	
	Soft palate	10	5 (50.0)		8	0 (0.0)	
	Pharyngeal wall	6	0 (0.0)		5	3 (60.0)	
Histology	MD SCC	43	19 (44.2)	0.331	36	7 (19.4)	0.527
	PD SCC	22	11 (50.0)		21	11 (52.4)	
	SCC NOS	30	17 (56.7)		25	7 (28.0)	
T stage	T1+T2	35	19 (54.3)	0.472	30	11 (36.7)	0.442
	T3+T4	60	28 (46.7)		52	14 (26.9)	
N stage	N0	23	14 (60.9)	0.208	21	5 (23.8)	0.441
	N+	72	33 (45.8)		61	20 (32.8)	
Composite stage	I+II	6	3 (50.0)	0.976	6	1 (16.7)	0.447
	III+IV	89	44 (49.4)		76	24 (31.6)	

The subsite was the only parameter that showed a significant difference concerning p16 positivity and HPV DNA positivity. The data on both p16 expression and HPV DNA status were available for 79 patients; however, no significant correlation was obtained (p=0.991).

Survival analysis

**Figure 1:**
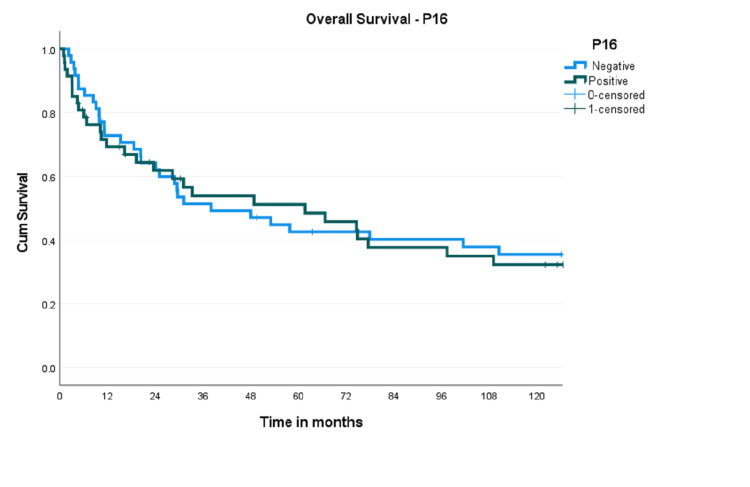
Kaplan-Meier curve showing overall survival probabilities of p16-positive and p16-negative tumours

The median follow-up was 134 months (1-160 months). The five-year attrition rate is 10.2%. The median survival in the whole population was 48 months (19.6-76.3), and the median time to recurrence was 17.2 months. The five-year OS probability was 42.6% (95% CI 28.49-56.71) and 51.2% (95% CI 35.92-66.48), respectively, for p16-negative and p16-positive tumours, p=0.689 (Figure 1).

**Figure 2:**
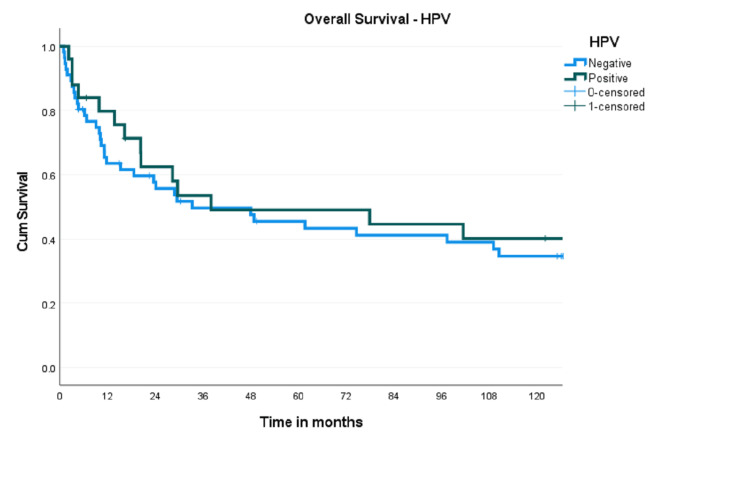
Kaplan-Meier curve showing overall survival probabilities of HPV DNA-positive and HPV DNA-negative tumours

The corresponding figures for five-year DFS were 49.0% (95% CI 34.7-63.3) and 51.9% (95% CI 36.62-67.18) (p=0.959). Survival analysis was also done based on HPV DNA status (both 16 and 18 subtypes together). The five-year OS for HPV-negative tumours was 45.5% (95% CI 32-59.02) compared to 49.1% (95% CI 28.72-69.48) in HPV-positive tumours (Figure 2).

Analysis based on HPV-16 DNA alone showed a 10-year OS of 31.9% (95% CI 19.75-44.05) for HPV-16-negative versus 54.2% (95% CI 29.12-79.28) for HPV-16-positive tumours (p=0.0309). The 10-year DFS also showed an absolute difference of 14%, favouring HPV-16-positive tumours (40.2%, 95% CI 27.27-53.13 versus 54.5%, 95% CI 29.42-79.58, p=0.234).

The survival probability was correlated with combined HPV and p16^INK4a^ expression status. Table 3 shows the five-year survival by HPV status and p16 expression.

**Table 3:** Overall survival and disease-free survival at five and 10 years by p16 expression and HPV DNA status OS: overall survival; DFS: disease-free survival; CI: confidence interval; HPV: human papillomavirus; DNA: deoxyribonucleic acid

Variables	Years	Overall Sample n=79	p16 Negative HPV Negative n=27	p16 Negative HPV Positive n=12	p16 Positive HPV Negative n=28	p16 Positive HPV Positive (SE) n=12	p-value
OS (95% CI)	5 years	47.5 (36.14-58.86)	44.4 (25.59-63.21)	41.7 (13.87-69.53)	48.6 (28.61-68.59)	63.5 (34.5-92.5)	0.817
	10 years	37.4 (25.84-48.96)	40.4 (21.8-59)	25.0 (5-45)	29.2 (10.19-39.39)	63.5 (34.49-92.50)	0.461
DFS (95% CI)	5 years	51.3 (39.94-62.66)	51.6 (32.59-70.61)	41.7 (13.87-69.53)	51.0 (31.8-70.2)	64.3 (35.69-92.9)	0.808
	10 years	47.9 (36.34-59.46)	51.6 (32.59-70.61)	33.3 (6.65-59.95)	44.6 (23.88-65.18)	32.1 (14.74-78.94)	0.766

The univariate Cox regression model, which is shown in Table 4, was based on p16 status and HPV DNA status and demonstrated no significant difference in the hazard ratio for death and recurrence.

**Table 4:** Univariate Cox regression model for mortality and recurrence CI: confidence interval; HR: hazard ratio; +ve: positive; -ve: negative; HPV: human papillomavirus

Variables	Mortality			Relapse		
	HR	95% CI	p-value	HR	95% CI	p-value
p16 Negative	1	-	0.890	1	-	0.959
p16 Positive	0.890	0.503-1.574		1.02	0.56-1.825	
HPV Negative	1	-	0.631	1	-	0.794
HPV Positive	0.848	0.433-1.662		0.913	0.463-1.803	
p16(-ve) & HPV(-ve)	1	-	-	1	-	-
p16(-ve) & HPV(+ve)	1.02	0.416-2.50	0.964	1.232	0.491-3.088	0.657
p16(+ve) & HPV(-ve)	0.957	0.45-2.01	0.909	1.146	0.531-2.473	0.729
p16(+ve) & HPV(+ve)	0.600	0.199-1.81	0.364	0.536	0.229-2.153	0.536

## Discussion

This is a retrospective study conducted at a tertiary cancer centre to study the prevalence and treatment response of oropharyngeal cancers for p16 and HPV status. The prevalence of HPV positivity (p16 or DNA) in oropharyngeal cancers ranges from 28% to 68% in Western literature [7,8]. A meta-analysis has reported overall pooled HPV prevalence in patients with OPSCC as 47.7% [9]. Indian data shows a decreased prevalence compared to the Western population, ranging from 20-30% [10,11]. Most of the published series are from North India. A prospective observational study from South India showed a prevalence of 26% [12]. The prevalence of p16 positivity in the present study is 49.47%, and HPV DNA positivity is 30.37%, both higher than the rates reported from South India. However, the rate of combined positivity is only 15.18%.

Several studies reported the correlation of p16 IHC with HPV DNA positivity [13]. The p16 expression and HPV DNA positivity did not show any correlation in the present study. Another Indian study similarly found no correlation between p16 and HPV DNA [14]. The lack of a significant correlation between p16 expression and HPV DNA positivity may be due to the small sample size. DNA PCR is best done in frozen tissue. However, in our study, paraffin-embedded tissue was used for DNA PCR. This could have resulted in lower sensitivity. In HPV DNA PCR, the transcriptional status cannot be ascertained, whereas p16 protein expression occurs after transcription. Moreover, the poor quality of the archived blocks would have influenced the interpretation of the results. Most of the literature reported HPV-16 as the main subtype responsible for OPSCC [7,15]. However, the distribution of HPV-16 and 18 subtypes was similar in this study.

Several studies have reported a higher prevalence of HPV-positive tumours in young patients [7,16]. Some recent trials showed an increasing incidence of HPV-positive OPSCC in older adults as well [17,18]. There was no difference in the median age at presentation among HPV-positive and -negative cohorts in the present study. The younger median age in the Western population may be due to the difference in culture and behaviour compared to the Indian population. Historically, most of the HPV-positive tumours were reported in males, likely associated with high-risk sexual behaviour [15]. This study also observed higher rates of p16 positivity among males. An increasing trend among women has been reported in the United States [18], and a meta-analysis has reported a similar prevalence of HPV-related OPSCC in males and females [19].

According to Ang et al., HPV-positive OPSCC were more common among nonsmokers and light smokers [7]. There are conflicting reports on the correlation between tobacco use and HPV positivity in India. Bahl et al. reported no impact of smoking and alcohol on HPV status, whereas a study from northeast India reported a significant association of high-risk HPV with tobacco chewing and alcohol consumption [11,20]. This study did not show any difference in p16 expression or HPV DNA positivity according to the smoking status, tobacco chewing, or alcohol intake.

The reason for the preference of HPV for the oropharynx is unclear; however, it may be related to the unique presence of nonkeratinised mucosa in the oropharynx, predominantly in the tonsillar tissue. The genetic features of HPV-16 may also facilitate survival in the tonsillar crypt epithelium [21]. Tonsil was the most common subsite to be affected by HPV infection in this series, similar to other published reports [15,22]. A recent systematic review of observational studies also showed that half of the tonsillar carcinomas were associated with HPV infection [23].

The expression of p16 is considered a surrogate marker for HPV-related oropharyngeal cancers. Several studies have reported improved survival in p16-positive OPSCC [24,25]. Although the present study showed an absolute benefit of 8.6% in the five-year OS in p16-positive patients, the difference was not statistically significant (51.2% versus 42.6%, p=0.689). Another study from India also reported similar survival in p16-positive and -negative patients [14]. There was no difference in survival concerning HPV DNA status (49.1%; 95% CI 28.72-69.48 versus 45.5%; 95% CI 32-59.0, p=0.631). This contradicts multiple studies that reported improved outcomes with HPV DNA positivity in OPSCC [7,8].

The majority of patients with p16 positivity or HPV DNA positivity were smokers in this study, which is different from the Western population, where p16/HPV positivity was higher in nonsmokers and light smokers. There is evidence that smoking is associated with poor outcomes, even in HPV-positive tumours [26]. There was an absolute difference of 20% in the five-year OS between double-positive and double-negative tumours in the present study. In a retrospective cohort study, Mena et al. came out with a similar observation that double positivity is associated with diagnostic accuracy and improved survival [27]. Two meta-analyses also supported improved outcomes in double-positive OPSCC [28,29].

Strengths and limitations

The study was conducted in a single institution with uniform treatment protocols. The sample size was low, and even though there were differences in the survival between HPV-positive and -negative tumours, statistical significance was not attained. Being a retrospective study with a limited sample size, the p16 and HPV DNA correlation and their association with outcomes should be interpreted with caution. The older paraffin blocks with limited tumour tissue archived from pathology may have resulted in false-positive or false-negative results. Additionally, there were many invalid PCR and IHC reports.

## Conclusions

Approximately 50% of the patients in this study demonstrated p16 positivity; however, the prevalence of HPV DNA positivity was lower. No significant correlation was observed between p16 expression and HPV status. Double positivity (p16 and HPV positive) was associated with better OS and DFS compared to double-negative (p16 and HPV negative) and single-positive (either p16 positive or HPV positive) cases. This subgroup of patients might benefit from potential de-escalation strategies and should be the target population for future studies. A prospective observational study with a larger sample size is warranted to identify the true prevalence of HPV and the potential correlation between p16 IHC and HPV DNA PCR and their influence on outcomes in the Indian population.

## References

[1] Bray F, Ferlay J, Soerjomataram I, Siegel RL, Torre LA, Jemal A (2018). Global cancer statistics 2018: GLOBOCAN estimates of incidence and mortality worldwide for 36 cancers in 185 countries. CA Cancer J Clin.

[2] Chaturvedi AK, Engels EA, Pfeiffer RM (2011). Human papillomavirus and rising oropharyngeal cancer incidence in the United States. J Clin Oncol.

[3] Boscolo-Rizzo P, Del Mistro A, Bussu F (2013). New insights into human papillomavirus-associated head and neck squamous cell carcinoma. Acta Otorhinolaryngol Ital.

[4] Kumar B, Cordell KG, Lee JS (2008). EGFR, p16, HPV Titer, Bcl-xL and p53, sex, and smoking as indicators of response to therapy and survival in oropharyngeal cancer. J Clin Oncol.

[5] D'Souza G, Kreimer AR, Viscidi R (2007). Case-control study of human papillomavirus and oropharyngeal cancer. N Engl J Med.

[6] Gillison ML (2008). Human papillomavirus-related diseases: oropharynx cancers and potential implications for adolescent HPV vaccination. J Adolesc Health.

[7] Ang KK, Harris J, Wheeler R (2010). Human papillomavirus and survival of patients with oropharyngeal cancer. N Engl J Med.

[8] Fakhry C, Westra WH, Li S (2008). Improved survival of patients with human papillomavirus-positive head and neck squamous cell carcinoma in a prospective clinical trial. J Natl Cancer Inst.

[9] Mehanna H, Beech T, Nicholson T, El-Hariry I, McConkey C, Paleri V, Roberts S (2013). Prevalence of human papillomavirus in oropharyngeal and nonoropharyngeal head and neck cancer—systematic review and meta-analysis of trends by time and region. Head Neck.

[10] Murthy V, Calcuttawala A, Chadha K (2017). Human papillomavirus in head and neck cancer in India: current status and consensus recommendations. South Asian J Cancer.

[11] Bahl A, Kumar P, Dar L (2014). Prevalence and trends of human papillomavirus in oropharyngeal cancer in a predominantly north Indian population. Head Neck.

[12] Anand AS, Presenavarman G, Windsor SR (2020). Therapeutic outcomes of HPV positive and HPV negative oropharyngeal squamous cell carcinomas. J Cancer Ther.

[13] Reimers N, Kasper HU, Weissenborn SJ (2007). Combined analysis of HPV-DNA, p16 and EGFR expression to predict prognosis in oropharyngeal cancer. Int J Cancer.

[14] Murthy V, Swain M, Teni T (2016). Human papillomavirus/p16 positive head and neck cancer in India: prevalence, clinical impact, and influence of tobacco use. Indian J Cancer.

[15] Schache AG, Powell NG, Cuschieri KS (2016). HPV-related oropharynx cancer in the United Kingdom: an evolution in the understanding of disease etiology. Cancer Res.

[16] Chaturvedi AK, Engels EA, Anderson WF, Gillison ML (2008). Incidence trends for human papillomavirus-related and -unrelated oral squamous cell carcinomas in the United States. J Clin Oncol.

[17] Windon MJ, D'Souza G, Rettig EM (2018). Increasing prevalence of human papillomavirus-positive oropharyngeal cancers among older adults. Cancer.

[18] Tota JE, Best AF, Zumsteg ZS, Gillison ML, Rosenberg PS, Chaturvedi AK (2019). Evolution of the oropharynx cancer epidemic in the United States: moderation of increasing incidence in younger individuals and shift in the burden to older individuals. J Clin Oncol.

[19] Mariz BA, Kowalski LP, William WN Jr (2020). Global prevalence of human papillomavirus-driven oropharyngeal squamous cell carcinoma following the ASCO guidelines: a systematic review and meta-analysis. Crit Rev Oncol Hematol.

[20] Kumar R, Rai AK, Das D (2015). Alcohol and tobacco increases risk of high risk HPV infection in head and neck cancer patients: study from north-east region of India. PLoS One.

[21] Joseph AW, D'Souza G (2012). Epidemiology of human papillomavirus-related head and neck cancer. Otolaryngol Clin North Am.

[22] Klussmann JP, Weissenborn SJ, Wieland U (2001). Prevalence, distribution, and viral load of human papillomavirus 16 DNA in tonsillar carcinomas. Cancer.

[23] Sethi S, Shahin A, Rahim IN (2024). Association of human papillomavirus infection with tonsillar cancers: a systematic review. Indian J Otolaryngol Head Neck Surg.

[24] Lewis JS Jr, Thorstad WL, Chernock RD, Haughey BH, Yip JH, Zhang Q, El-Mofty SK (2010). p16 positive oropharyngeal squamous cell carcinoma: an entity with a favorable prognosis regardless of tumor HPV status. Am J Surg Pathol.

[25] Rischin D, Young RJ, Fisher R (2010). Prognostic significance of p16INK4A and human papillomavirus in patients with oropharyngeal cancer treated on TROG 02.02 phase III trial. J Clin Oncol.

[26] Grønhøj C, Jensen JS, Wagner S (2019). Impact on survival of tobacco smoking for cases with oropharyngeal squamous cell carcinoma and known human papillomavirus and p16-status: a multicenter retrospective study. Oncotarget.

[27] Mena M, Taberna M, Tous S (2018). Double positivity for HPV-DNA/p16(ink4a) is the biomarker with strongest diagnostic accuracy and prognostic value for human papillomavirus related oropharyngeal cancer patients. Oral Oncol.

[28] Albers AE, Qian X, Kaufmann AM, Coordes A (2017). Meta analysis: HPV and p16 pattern determines survival in patients with HNSCC and identifies potential new biologic subtype. Sci Rep.

[29] Coordes A, Lenz K, Qian X, Lenarz M, Kaufmann AM, Albers AE (2016). Meta-analysis of survival in patients with HNSCC discriminates risk depending on combined HPV and p16 status. Eur Arch Otorhinolaryngol.

